# A Rare Case Report of Dengue Fever Presenting With Acute Leukoencephalopathy With Restricted Diffusion

**DOI:** 10.7759/cureus.74835

**Published:** 2024-11-30

**Authors:** Soham N Naik

**Affiliations:** 1 Radiodiagnosis, Sir Takhtasinhji General Hospital, Bhavnagar, IND

**Keywords:** adem, alerd, anec, dengue, encephalopathy, neuroradiology, seizure, viral encephalitis

## Abstract

Acute leukoencephalopathy with restricted diffusion (ALERD) is an emerging clinico-radiological syndrome marked by sudden onset encephalopathy and characteristic restricted diffusion in the subcortical white matter on MRI. While typically linked to other viral etiologies, its association with dengue fever is not well known and is rarely documented in the literature. In this report, we describe a rare case of ALERD associated with dengue fever in a young patient, emphasizing clinical features, neuroimaging findings, management, and outcome. This case reinforces the need to consider dengue as a potential etiologic factor for ALERD in endemic regions and highlights the importance of timely intervention for improving patient outcomes.

## Introduction

Acute leukoencephalopathy with restricted diffusion (ALERD) is a clinical syndrome characterized by acute encephalopathy and specific MRI findings of restricted diffusion in the subcortical white matter, often observed within a few days of symptom onset [1]. Although initially associated with infectious and toxic etiologies, the list of potential triggers for ALERD has expanded.

Dengue fever, a viral infection endemic to tropical and subtropical regions, is most commonly associated with systemic symptoms, including high-grade fever, thrombocytopenia, hepatomegaly, and hemorrhagic manifestations. However, neurological complications such as dengue encephalitis, myelitis, and acute disseminated encephalomyelitis (ADEM) have been increasingly recognized [2]. Dengue encephalitis typically presents with nonspecific findings on MRI, including cerebral edema, hyperintensities in the thalamus, basal ganglia, and brainstem, and, occasionally, focal hemorrhages in these areas [3]. Such findings are usually consistent with viral encephalitis and reflect the systemic nature of dengue’s impact on the CNS.

In contrast, ALERD represents a rare, distinct syndrome characterized by acute encephalopathy and specific MRI findings of restricted diffusion in the subcortical white matter, typically without hemorrhage. The syndrome has been associated with infectious triggers, particularly in pediatric patients, and is most often reported in cases linked to influenza and herpes viruses [1]. ALERD has a characteristic radiological pattern of restricted diffusion, usually presenting in either a “diffuse” form associated with severe clinical courses or a “central sparing” form, which is often milder [4].

ALERD has not been commonly reported as a neurological complication of dengue fever, and its presentation in this context is rare, possibly due to the unique pathophysiological interactions between the dengue virus and the central nervous system. This case report discusses a rare instance of ALERD triggered by dengue fever in a pediatric patient, providing insight into its unusual presentation, clinical course, and outcomes.

## Case presentation

A 16-month-old male child presented to the emergency department in the Department of Pediatrics at Sir Takhtasinhji General Hospital with a five-day history of high-grade fever (39-40°C) and multiple episodes of vomiting. His symptoms had progressed, with altered consciousness and a generalized tonic-clonic seizure occurring on the fifth day of illness. Physical examination upon admission revealed a stuporous child with a Glasgow Coma Scale (GCS) score of 10/15, bilateral conjunctival injection, and mild hepatomegaly. No rash or overt signs of hemorrhage were noted, though he had evidence of diffuse petechiae on closer inspection.

Investigations and laboratory findings

Initial laboratory tests were significant for elevated liver enzymes (AST: 1300 IU/L, ALT: 850 IU/L), severe thrombocytopenia (platelet count: 45,000/mm³), and elevated prothrombin time (INR: 1.7), which are markers consistent with severe dengue. His serum dengue IgM and NS1 antigen tests returned positive, confirming an acute dengue infection. The patient also had elevated serum lactate levels (4.5 mmol/L), indicating possible lactic acidosis secondary to shock.

Cerebrospinal fluid (CSF) analysis showed normal cell counts, normal protein, and glucose levels, with no pathogens detected on PCR testing for viral encephalitis panel (including HSV, CMV, and enteroviruses). Electroencephalography (EEG) showed diffuse cerebral dysfunction but no overt epileptiform activity. Blood and urine cultures were negative, ruling out bacterial co-infections. Renal function tests remained within normal limits, and chest radiography was unremarkable.

Neuroimaging findings

**Figure 1:**
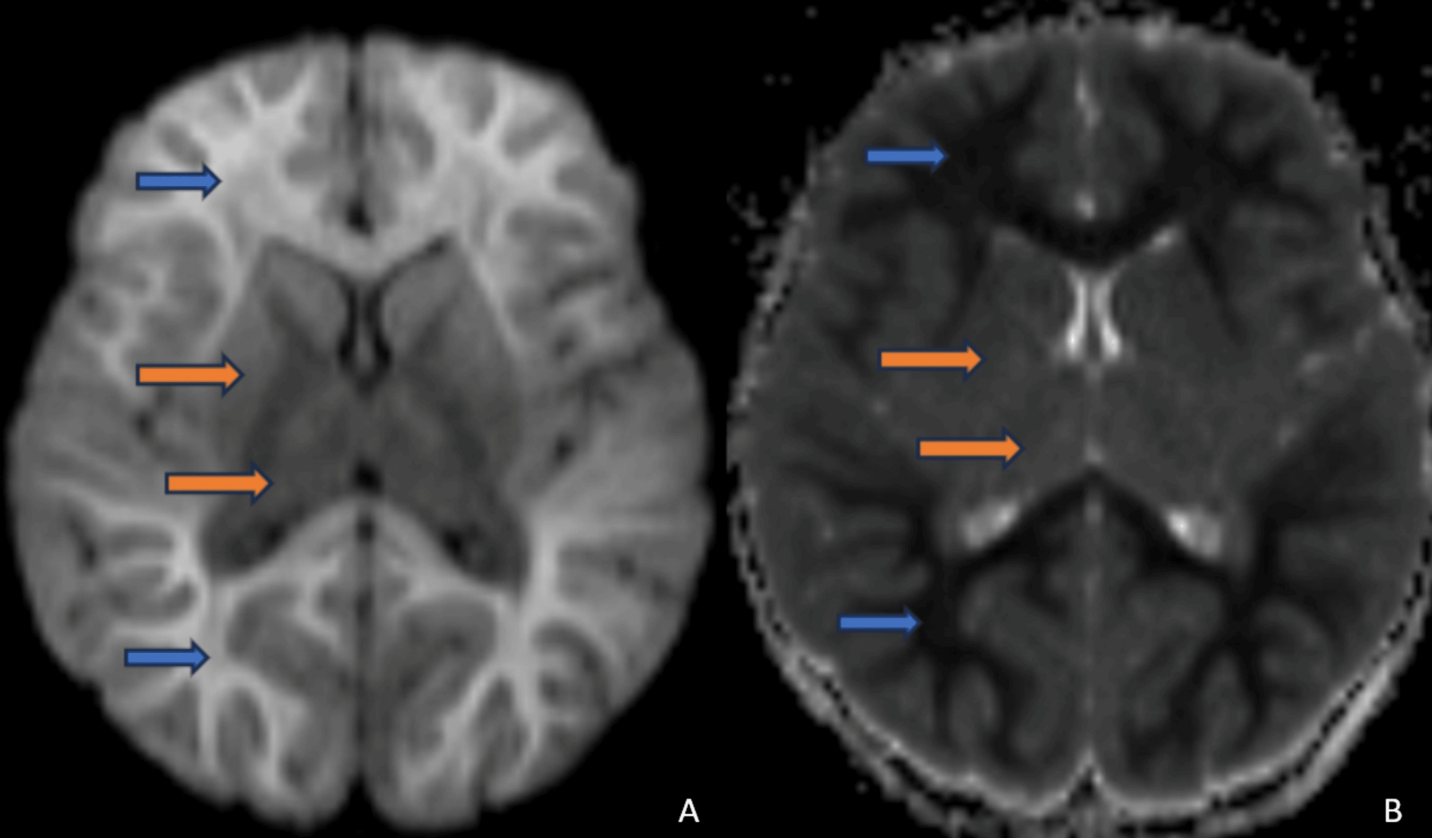
Diffusion weighted images and corresponding ADC images show marked diffusion restriction in form of high signal on DWI [1A] with corresponding low ADC value [1B] in subcortical white of bilateral frontal and parietal lobes (Blue arrow). The basal ganglia and thalamus are spared (orange arrow). ADC: Apparent diffusion coefficient, DWI: Diffusion weighted imaging

**Figure 2:**
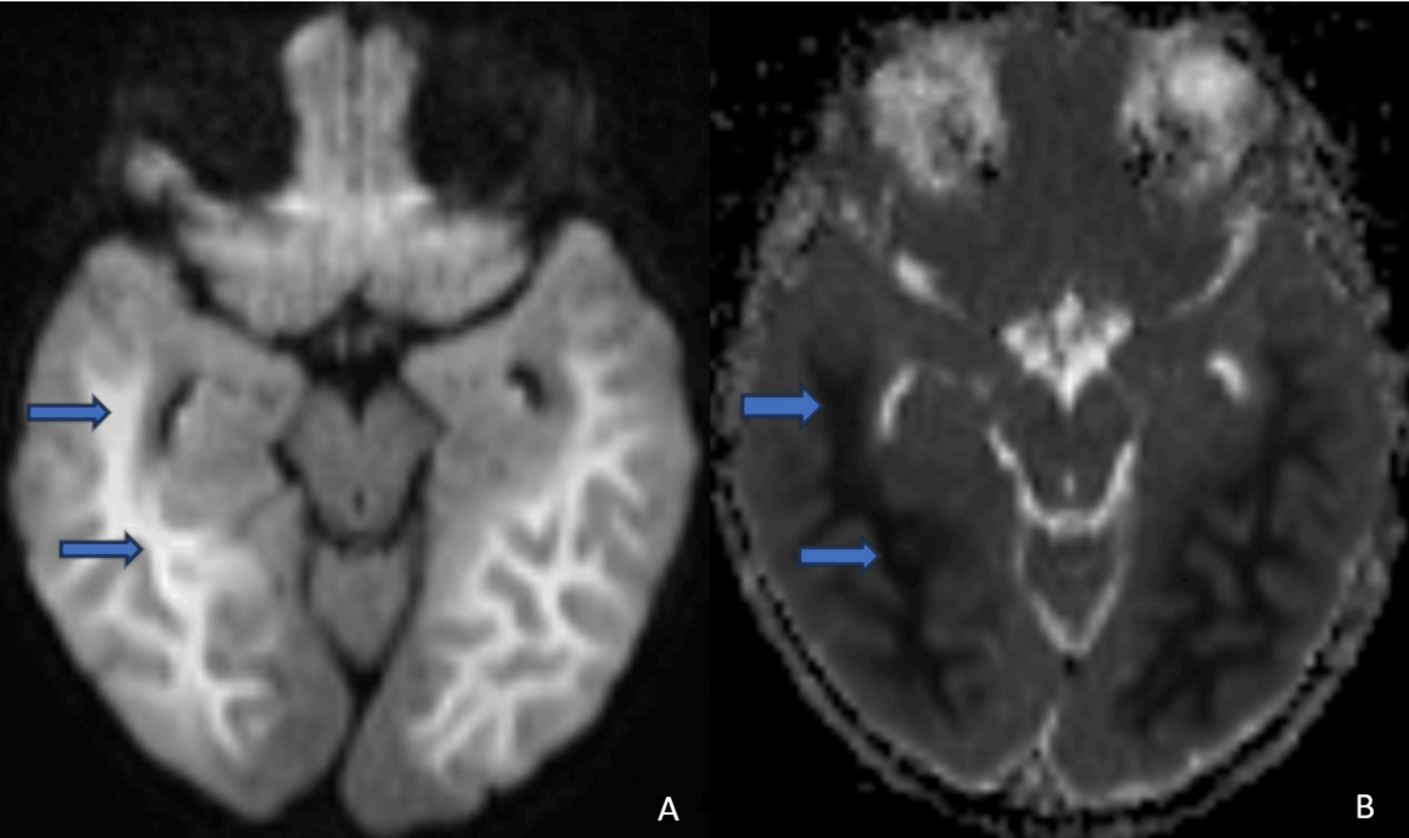
Diffusion weighted images (DWI) and corresponding ADC images show marked diffusion restriction in the form of a high signal on DWI [2A] and a corresponding low signal on ADC [2B] in the subcortical white of bilateral temporal and occipital lobes (blue arrow). ADC: Apparent diffusion coefficient, DWI: Diffusion weighted imaging

**Figure 3:**
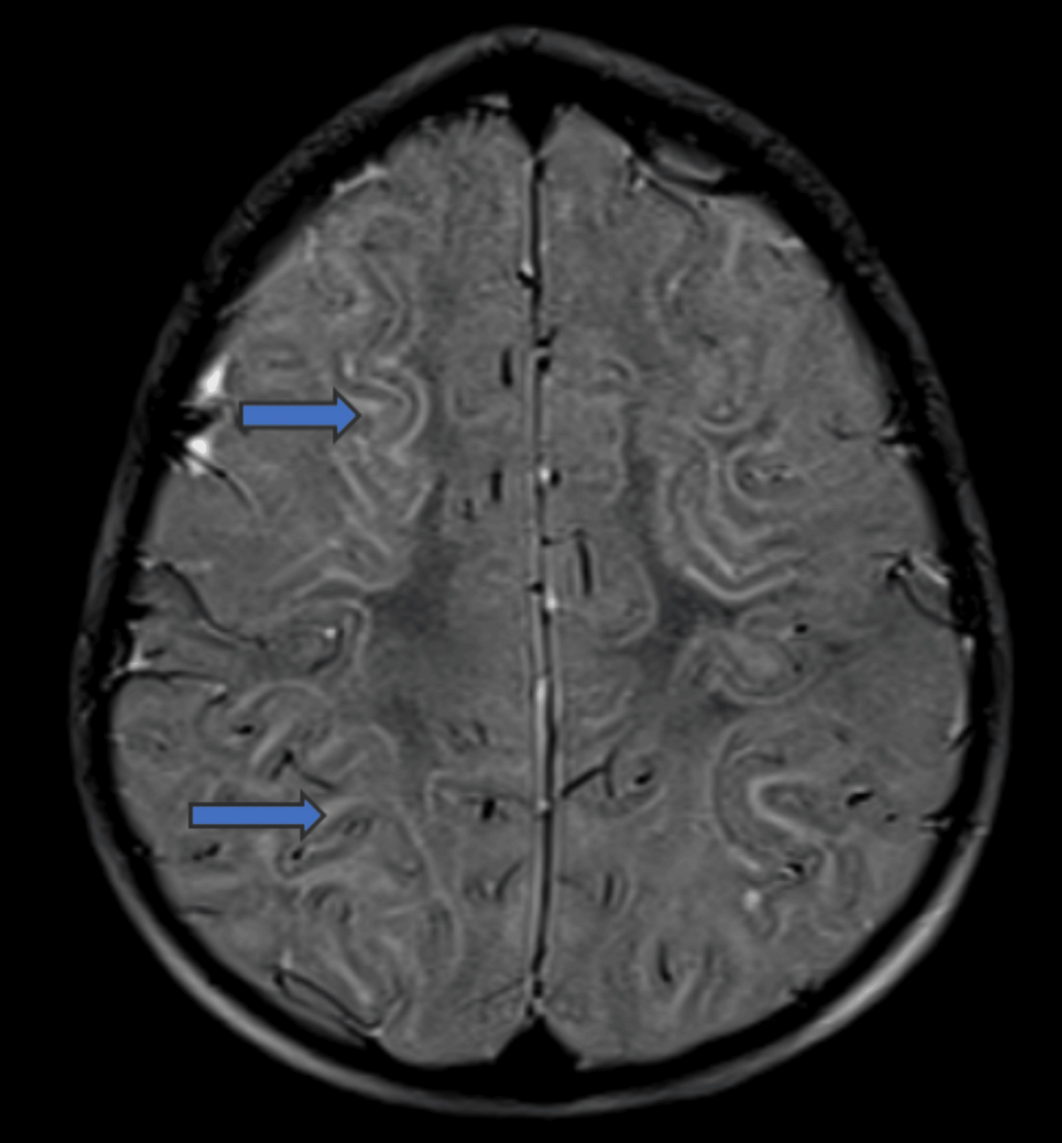
T2 weighted image demonstrates hyperintense signal intensity in subcortical white matter of bilateral fronto-parietal lobes.

Magnetic resonance imaging (MRI) of the brain was performed on day six of the illness and revealed characteristic findings of ALERD. Diffusion-weighted imaging (DWI) showed bilateral symmetrical areas of restricted diffusion, predominantly in the subcortical white matter, without central sparing in bilateral frontal and parietal lobes (Figures 1A, 1B) and bilateral temporal and occipital lobes (Figures 2A, 2B). T2-weighted images demonstrated corresponding mild hyperintense signals in the affected areas, indicative of cytotoxic edema (Figure 3). The basal ganglia and thalamus were spared, and no hemorrhage or necrosis was observed (Figures 1A, 1B). This diffuse pattern of restricted diffusion is typically associated with more severe presentations of ALERD and correlated with the patient’s deteriorating neurological status.

Management

The patient was managed in the pediatric intensive care unit with comprehensive supportive care. Standard management for severe dengue was initiated, including aggressive intravenous fluid resuscitation, strict monitoring of electrolyte balance, and platelet transfusions as needed to manage coagulopathy.

In light of the diagnosis of ALERD, the patient received high-dose intravenous methylprednisolone for five days to address possible inflammation and immune response associated with dengue. Also, intravenous immunoglobulin (IVIG) was administered over 48 hours to mitigate potential cytokine-mediated damage. Anticonvulsant therapy with levetiracetam was continued to prevent further seizures, though no additional seizure activity was observed following initial control.

Outcome and Follow-up

**Figure 4:**
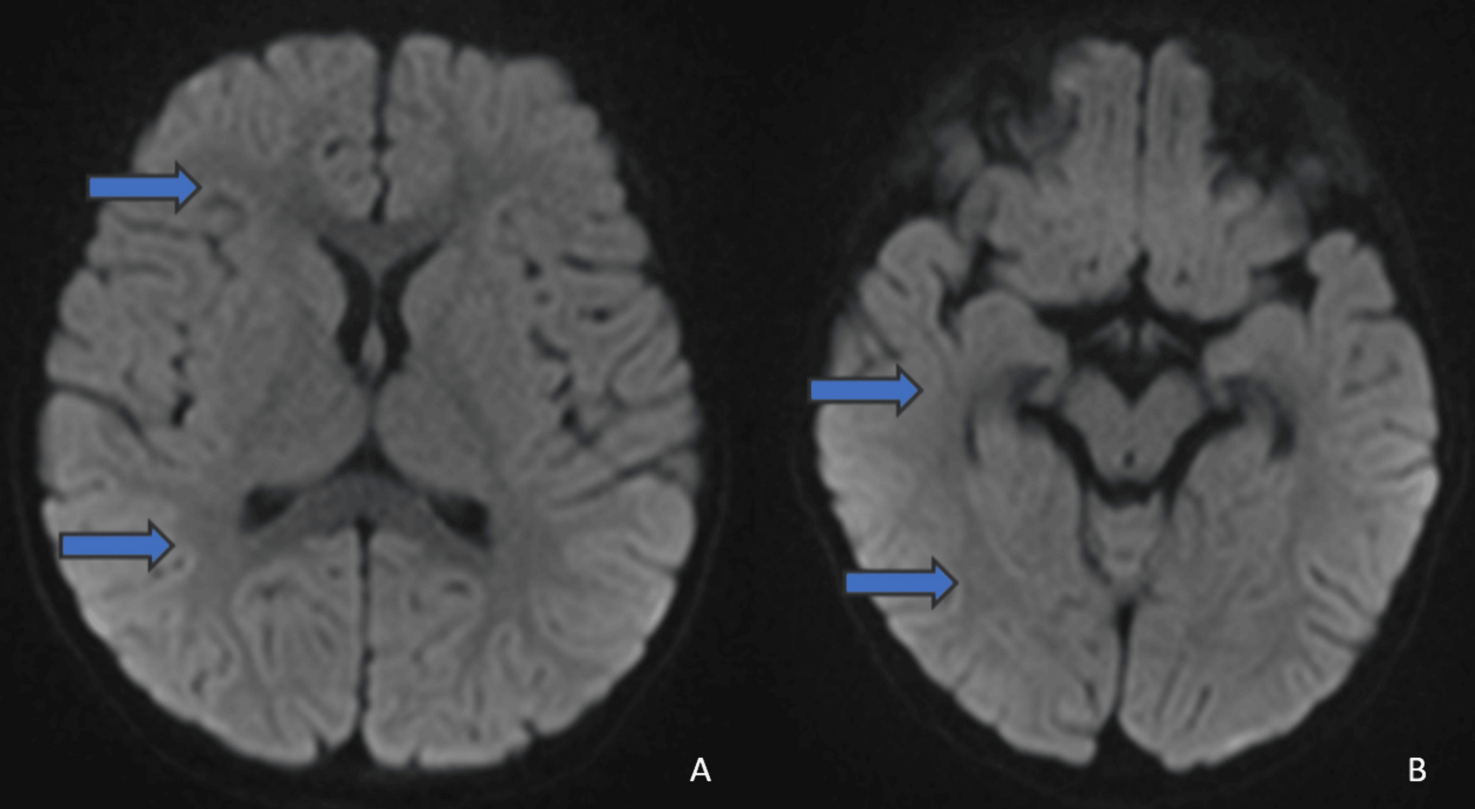
Diffusion-weighted images on day 14 show no high signal on DWI images in bilateral frontal and parietal lobes [4A] as well as bilateral temporal and occipital lobes [4B]. DWI: Diffusion weighted imaging

The patient’s clinical condition improved steadily over his hospitalization. Over the next 12 days, he demonstrated gradual recovery of cognitive and motor functions. Neuroimaging follow-up on day 14 showed a marked reduction in the areas of restricted diffusion, aligning with his clinical recovery (Figures 4A, 4B). By day 16, he had fully recovered, demonstrating normal cognitive and motor function. He was subsequently discharged with no significant neurological deficits.

At the one-month follow-up, the patient exhibited no significant cognitive deficits, with normal performance in attention, memory, and executive functions. This favorable outcome highlights the importance of early recognition and treatment in dengue-associated ALERD, particularly in severe cases.

## Discussion

Dengue fever is predominantly a systemic disease, but CNS involvement is increasingly recognized, with presentations ranging from mild encephalopathy to severe forms like ALERD [2]. The pathogenesis of dengue-induced ALERD remains unclear; however, it may involve immune-mediated mechanisms, direct viral invasion, or secondary effects of the cytokine storm often seen in severe dengue [1]. The neuroimaging findings in ALERD are distinct and play a crucial role in differentiating it from other acute encephalopathic syndromes, such as acute disseminated encephalomyelitis (ADEM) and acute necrotizing encephalopathy (ANE), which may also present with altered consciousness, seizures, and other neurologic signs [5].

ALERD is characterized by restricted diffusion on MRI, predominantly in the subcortical white matter, with involvement varying from diffuse patterns to more centrally sparing patterns. Diffuse involvement, as seen in this case, generally suggests a more severe presentation, with cytotoxic edema and a worse clinical prognosis [6]. Restricted diffusion in ALERD reflects cytotoxic edema due to cellular injury, often without concurrent hemorrhage or necrosis, differentiating it from other conditions with subcortical involvement.

ADEM is an immune-mediated demyelinating disorder often triggered by infections or vaccinations, predominantly affecting the white matter [7]. Unlike ALERD, which is characterized by cytotoxic edema, ADEM exhibits inflammatory demyelination. MRI findings in ADEM typically include multifocal, asymmetric hyperintensities on T2-weighted and FLAIR images, primarily in the deep and subcortical white matter. In some cases, the basal ganglia, thalamus, and brainstem may also be involved, but restricted diffusion is uncommon in ADEM, which helps distinguish it from ALERD. Additionally, contrast enhancement is often present in ADEM lesions, suggesting a breakdown of the blood-brain barrier, an inflammatory hallmark not usually seen in ALERD.

While both ADEM and ALERD may present with altered consciousness and neurologic deficits, the imaging findings help differentiate them. ADEM's characteristic demyelinating lesions, often accompanied by gadolinium enhancement, are generally larger, multifocal, and lack the distinct restricted diffusion seen in ALERD.

ANE, another rare but severe encephalopathic condition often triggered by dengue [8], is characterized by bilateral, symmetrical necrotic lesions, particularly involving the thalami, brainstem, and, occasionally, the cerebral white matter. Unlike ALERD, ANE is distinguished by its characteristic necrosis and the presence of hemorrhagic changes on imaging. MRI in ANE typically shows bilateral thalamic involvement with areas of hyperintensity on T2-weighted images, sometimes with a “halo” of restricted diffusion around necrotic regions. Hemorrhagic components are common and can be detected on susceptibility-weighted imaging (SWI) or gradient-echo sequences.

The presence of hemorrhage, necrosis, and involvement of deep gray matter structures in ANE provides a clear distinction from ALERD, where restricted diffusion is limited to the subcortical white matter and rarely involves the thalami or brainstem. The absence of hemorrhagic features in ALERD further supports the differentiation, as ALERD-associated changes are typically non-hemorrhagic, indicating a different pathophysiological mechanism.

The neuroimaging patterns in ADEM, ANE, and ALERD underscore different underlying mechanisms. Cytokines play a pivotal role in the neurological manifestations and varying neuroimaging patterns observed in dengue fever. In conditions like ALERD, the cytokine storm triggered by the dengue virus leads to widespread inflammation and endothelial dysfunction, resulting in cytotoxic edema. This is reflected as restricted diffusion predominantly in the subcortical white matter on MRI. Conversely, in dengue encephalitis, elevated levels of pro-inflammatory cytokines such as TNF-α and IL-1β can contribute to more generalized cerebral edema and hyperintensities in the thalamus and brainstem, often without restricted diffusion. Furthermore, ALERD lacks the multifocal inflammatory demyelination seen in ADEM and the characteristic necrosis and hemorrhage in the thalami typical of ANE, making its imaging and clinical course distinct. These differences highlight the importance of recognizing ALERD as a separate clinico-radiologic entity, particularly in the context of infectious triggers like dengue fever, which may elicit a unique form of CNS involvement not seen in ADEM or ANE.

Factors influencing prognosis in ALERD include duration of seizure, presence of status epilepticus, and hepatic involvement [9]. The presence of multiorgan dysfunction, as observed in our patient with significant liver involvement and coagulopathy, underscores the complex interplay between dengue fever and ALERD.

Treatment for ALERD remains largely supportive, though corticosteroids and immunoglobulins have been used to manage inflammatory responses in severe cases [4]. Although prospective studies on treatment efficacy are lacking, our case suggests that early intervention with steroids and IVIG may support improved outcomes in dengue-associated ALERD, as evidenced by the gradual recovery observed in this patient.

## Conclusions

This case highlights a rare but significant neurological complication of dengue fever, presenting as acute leukoencephalopathy with restricted diffusion (ALERD). In regions where dengue is endemic, clinicians should remain vigilant for neurological symptoms, particularly in severe cases with multi-organ involvement. Early recognition of imaging findings and intervention may improve outcomes, though further studies are needed to establish specific treatment protocols for dengue-associated ALERD. Continued research into the pathophysiological mechanisms of ALERD and its relationship with infectious triggers like dengue may eventually provide insights into more targeted and effective therapies for this syndrome.
